# Circulating non-coding RNAs as potential diagnostic biomarkers in liver diseases 

**Published:** 2021

**Authors:** Fedra Mokhtari, Seyed Reza Mohebbi, Afsaneh Sharifian, Marzieh Ramandi, Mohammad Reza Razzaghi

**Affiliations:** 1 * Basic and Molecular Epidemiology of Gastrointestinal Disorders Research Center, Research Institute for Gastroenterology and Liver Diseases Shahid Beheshti University of Medical Sciences, Tehran, Iran*; 2 * Gastroenterology and Liver Diseases Research Center, Research Institute for Gastroenterology and Liver Diseases, Shahid Beheshti University of Medical Sciences, Tehran, Iran*; 3 *Proteomics Research Center, Faculty of Paramedical Sciences, Shahid Beheshti University of Medical Sciences, Tehran, Iran*; 4 *Laser Application in Medical Sciences Research Center, Shahid Beheshti University of Medical Sciences, Tehran, Iran*

**Keywords:** Non-coding RNAs, microRNAs, Circulating biomarkers, Liver diseases, Viral hepatitis, Chronic disease

## Abstract

The liver plays a principal role in the human body as a metabolic and detoxifying unit. Liver diseases are the world’s major health problems and affect millions of people worldwide. Early detection of liver diseases is certainly effective in timely treatment and prevention of their progression. Liver injury is associated with significant alterations in immune responses and pattern changes in various tissue-related gene expressions and cytokine production. Increasing or decreasing the specific spectrum of non-coding RNAs in different phases of liver disease can be a criterion for diagnosis. Novel diagnostic biomarkers are needed for liver diseases. Currently, micro-RNAs (miRNAs) are known to play important roles in the diagnosis of liver diseases. Circulating biomarkers such as miRNA-assisted diagnosis can conceivably be helpful for the early treatment of liver diseases. In this review, we look at miRNAs and their potential applications in liver diseases as diagnostic biomarkers were investigated.

## Introduction

**Figure 1:**
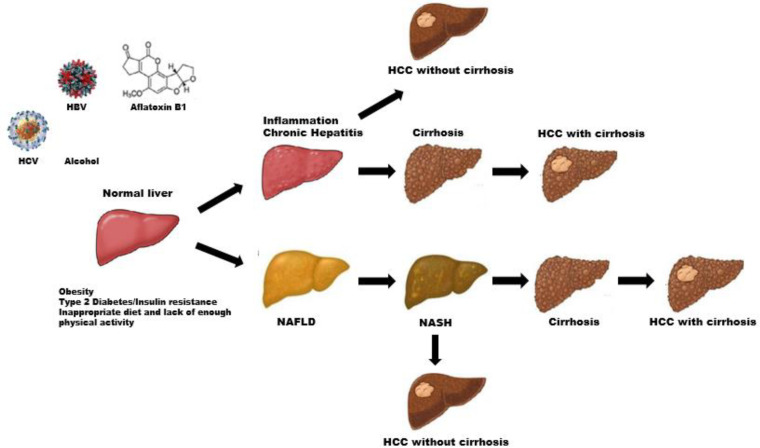
Etiologies and natural history and progression of chronic liver diseases. HBV: Hepatitis B virus, HCV: Hepatitis C virus, NAFLD: nonalcoholic fatty liver disease, NASH: nonalcoholic steatohepatitis, HCC: hepatocellular carcinoma

 Liver diseases are common causes of mortality in the world and comprise different types such as hepatitis A, B, C and D (acute and chronic), cirrhosis, autoimmune related liver diseases including autoimmune hepatitis (AIH), primary biliary cirrhosis (PBC), primary sclerosing cholangitis (PSC), nonalcoholic fatty liver (NAFLD), nonalcoholic steatohepatitis (NASH), alcoholic steatohepatitis (ASH), and hepatocellular carcinoma (HCC). Liver damage occurs in different stages (Fig. 1). Miscellaneous factors can injure the liver, including viral infections, toxins, hereditary conditions, or autoimmune processes; however, the main etiologies include viral infections, alcohol consumption, and exposure to high levels of some toxic chemicals. 

Clinical data shows that there are up to 400 million HBV surface antigen carriers worldwide (1), and about 180 million people are infected with the hepatitis C virus (HCV) (2). Approximately 170,000 deaths due to liver cirrhosis occur annually in Europe (3). Moreover, evidence indicates liver cirrhosis-related mortality has escalated during recent years. In the United States, it has been estimated that between 0.15% in 1998 to 0.27% in 2010 (1) of people deal with NAFLD (4). Distinct types of liver diseases in different stages place a heavy economic and psychological burden on governments and societies, and therefore, the precise identification and diagnosis of diseases at different stages are very important. MicroRNA (miRNA) is a small-sized non-coding RNA that binds to the target mRNA, resulting in mRNA degradation or translation inhibition. miRNAs play important roles in regulating cell growth, proliferation, and metabolism. Circulating miRNAs have been studied as a candidate for diagnosis of a variety of diseases, such as malignancies and cardiovascular, neurologic, or metabolic diseases, including diabetes, NAFLD, and other liver diseases (2).

**Liver disease in the Middle East**

Liver and gastrointestinal diseases are also major causes of health problems and deaths in the Middle East (6, 7), with liver cirrhosis being one of the top four causes of mortality in the Middle East. However, there is a significant heterogeneity in the pattern, incidence, mortality, and burden of gastrointestinal and liver diseases in the region. Infectious diseases causing gastrointestinal (GI) and liver diseases still remain highly important in countries like Pakistan and Afghanistan; on the other hand, a clear shift from contagious to non-communicable GI diseases is noticeable in countries with a more structured healthcare system and higher economical levels. Pakistan has experienced a significant rate of cirrhosis. Mortality caused by cirrhosis in Egypt has also been significantly high, followed by other regional countries including Afghanistan, Yemen, and Morocco. Chronic liver diseases are very prevalent in Iraq with the most important causes being hepatitis B, hepatitis C, immune hepatitis, and metabolic diseases, in rank order. Non-alcoholic fatty liver disease has shown an increasing rate, and non-alcoholic steatohepatitis became a leading indication for liver transplantation in this country. In Saudi Arabia, Kuwait, the United Arab Emirates, Qatar, Bahrain, Oman, Jordan, and Iraq, cases of GI cancers are increasing, and chronic hepatitis B and C are the main causes of liver cirrhosis and progression to hepatocellular carcinoma (8-11). Around 25% of the global adult population, ranging from 13.5% in Africa to 31.8% in the Middle East, experience NAFLD complications (3). Additionally, the worldwide burden of HCC mortality is anticipated to hit one million deaths annually by 2030 (12-14).

In Iran, the consequences of cancers of the stomach and esophagus are significantly greater than other countries. The Global Burden of Disease (GBD) study showed that almost 5400 deaths due to chronic liver diseases such as cirrhosis occurred in Iran in 2017 (12, 13). In accordance with the recent GBD record from Iran, the predominant etiology of cirrhosis was NASH, with a prevalence of almost 18 million cases and an age-standardized prevalence rate of 20,500 per 100,000. Because of the effective national vaccination, the HBV prevalence has diminished during recent years (13, 15-17). HCV chronic infection rate in Iran is calculated to be 3-5 patients per 1,000 in the general population. However, HCV treatment has changed this data, with new direct acting antiviral drugs being produced by local pharmaceutical companies at an acceptable cost (18, 19). NAFLD has recently become a relatively usual etiology of chronic liver disease in Iran. It seems that a sedentary lifestyle and increased caloric intake are the main risk factors; however, genetic variations have been responsible in almost half of the patients, especially those with lean NAFLD. Viral hepatitis has been largely controlled, but NAFLD has risen as a noticeable problem to the health of the general society both in Iran and worldwide (20-23). 

Socioeconomic status and healthcare systems play important roles in the prevalence of different liver diseases in a population. Other factors, mainly those related to the host, may link to susceptibility to liver diseases, including HCV or HBV chronic infections. For example, genetic variations such as single nucleotide polymorphism in cytokine genes are probably associated with susceptibility to HCV or HBV chronic diseases (24-27). 

**Liver diseases**

**Viral hepatitis**

Hepatitis A, B, D, E, and occasionally hepatitis C infections may cause acute diseases of the liver. Hepatitis A virus (HAV) is a small RNA virus belonging to the Picornaviridae family that only leads to an acute hepatitis. It is transmitted through the fecal-oral route, while the hepatitis B virus (HBV) is transmitted parenterally (from mother to child) as well as sexually (28). HBV is a DNA virus and a member of the Hepadnaviridae family that is unique in its genomic characterization among human viruses, antigenic structure, and replication cycle (29). In addition to acute hepatitis, HBV can also lead to chronic liver disease. The hepatitis C virus (HCV) is a member of the Flaviviridea family and a blood-borne RNA virus (30). The hepatitis E virus (HEV) is also a small RNA virus that belongs to the Hepeviridea family and mainly causes acute hepatitis; however, chronic infections have been reported in patients with immune deficiency and transplant recipients (31, 32). HBV and HCV chronic infections can progress to liver cirrhosis in as many as 25% of cases and to hepatocellular carcinoma (HCC) in a significant proportion of infected patients (33). The hepatitis delta virus (HDV) is a small subviral particle with RNA genome. It can only infect HBV carriers. Hepatitis D tends to be a fierce disease, with an intense desire to lead to cirrhosis (34).

**Autoimmune liver diseases**

When the body’s immune system attacks the liver, autoimmune complications of the liver will occur. Nevertheless, with regard to grade of intensity and clinical course, these diseases can have different clinical patterns and outcomes. The most prominent autoimmune liver diseases (AILDs) can refer to autoimmune hepatitis (AIH), primary biliary cirrhosis (PBC), and primary sclerosing cholangitis (PSC). The target of the immune system in AIH is hepatocytes, and this consequently lead to chronic inflammation of the liver (34-36). In PBC, the autoimmune reactions are directed at small biliary ducts inside the liver. In primary sclerosing cholangitis, uncontrolled autoimmune responses target the larger extrahepatic bile ducts (37, 38). Several classes of autoantibodies are related to autoimmune liver diseases, such as AMA, ANA, anti-LKM, anti-SMA/anti-F-actin, and others. For AIH and PBC, detecting these autoantibodies is an effective prerequisite for diagnosis, but not for PBS, because their diagnostic value has not been well established. In spite of all that, diagnosis at an early stage can lead to hepatitis being controlled by proper treatments for suppressing inflammation; these treatments, however, are just for controlling and not curing. Genetic factors are strongly associated with the pathogenesis of AILDs. For example, like other autoimmune diseases, HLA loci is involved in the development of AIH and PBC (39, 40). A large cohort study has confirmed HLA-DRB1*0301 as a primary susceptibility genotype and HLA-DRB1*0401 as a secondary susceptibility genotype for AIH (41). Other alleles such as HLADRB1*0405, HLA-DRB1*0404, HLA-DRB1*07 and HLA-DRB1*13 alleles have been reported to increase the risk of developing AIH. Some studies have shown that HLA-DRB1*0803, HLA-DRB1*0801, HLA-DRB1*14, and HLADPB1*0301I are associated with PBC risk (42-47). According to studies, some non-HLA genes are susceptible to AIH, including CARD10, STAT4, SH2B3, CTLA-4, and TNFA. Moreover, other non-HLA genes responsible in signaling pathways can be considered as allelic risk factors of PBC, such as IL-12 production and antigen presenting process (e.g., IRF5, NF-kB, TNFAIP3, NF-kB, SOCS1, and IL-12A), T cell stimulating and secretion of IFN-γ (e.g., IL12R, TNFSF15, TYK2, TNFAIP3, and STAT4). In PBS, several susceptibility genes may play a role in bile acid homeostasis, such as HDAC7 and TGR5, and T cell-related risk loci (e.g., CTLA4/CD28, MMEL1/TNFRSF14, CD226, PRKD2, IL2/IL21, and CCL20) is associated with disease progression. Immune homeostasis may be disrupted by regulating immune tolerance, cytokine production, and immune responses due to dysfunctional gene products (48-51). However, the details of AILD development mechanisms remain unclear, while researchers have focused on epigenetic processes and microRNAs functions (52).

**NAFLD/NASH**

Nonalcoholic fatty liver has become a common cause of chronic liver disease. It is a range of conditions, from steatosis to nonalcoholic steatohepatitis (NASH), that can progress to fibrosis, cirrhosis, and hepatocellular carcinoma. The global prevalence of NAFLD is estimated at approximately 24%, with considerable variability from Africa (13.5%) to South America (30.5%), the Middle East (31.8%), and other regions of Asia (33.9%). Some factors can be considered causes for NAFLD, such as obesity, type 2 diabetes mellitus (T2DM), insulin resistance, and possibly iron metabolism problems. Leite et al. studied 180 T2DM patients and observed that 69%, 54%, and 28% of these patients also suffered from NAFLD, NASH, and liver fibrosis grade 2 or higher, respectively (53-59). Diabetes can aggravate NAFLD and lead to more severe forms of steatohepatitis, fibrosis, and even HCC. NAFLD itself can also modify the natural history of T2DM (60). Finally, in patients with T2DM, the presence of NAFLD is associated with an increased risk of overall death. Moreover, it is an independent predictor of death together with the presence of ischemic heart disease and duration of diabetes. The effects of NAFLD expand beyond the liver, and it is associated with a range of chronic diseases, especially cardiovascular disease (CVD), diabetes mellitus type 2 (T2DM), and chronic kidney disease (CKD). Fatty liver disease caused primarily by persistent excessive alcohol consumption is known as alcoholic liver disease (ALD). Differentiating between ALD and NAFLD is difficult, as it relies on patient-reported consumption (61). As obesity is common, risk factors for NAFLD may be present in individuals who also consume high levels of alcohol (62, 63). 

NASH is the progressive form of NAFLD (64). NASH may happen because of fat aggregation in the liver followed by hepatocellular ballooning, liver cell inflammation, and injury (steatohepatitis). NASH symptoms mainly consist of non-specific disease manifestations such as fatigue, abdominal pain, lethargy, and sleeping problems; thus, they may sometimes be falsely interpreted (65). Most people live with the disease for years unaware of the damage accumulating in their liver, because NASH is, typically, only detected when it has progressed to cirrhosis or liver cancer (66). NASH is defined as a series of necroinflammatory processes, whereby the liver cells become injured in a background of steatosis. The largest study to date to analyze the prevalence of NAFLD and fibrosis due to NASH was performed in a group of young, largely asymptomatic Caucasians with suspected NAFLD in the UK. The results revealed that one in five of the studied subjects had steatosis, and one in 40 showed evidence of fibrosis due to NASH at only 24 years of age (67). An increased risk of NAFLD and NASH at a younger age may progress and lead to serious health complications in early adulthood (68). People with NASH have an overall mortality rate of 7.9% within seven years of diagnosis (69).

**Cirrhosis and hepatocellular carcinoma**

Cirrhosis is the end phase of chronic liver disease, and fibrosis is the stage prior to cirrhosis (70). Many types of molecules such as miRNAs and cytokines are associated with the start and advancement of liver fibrosis and, therefore, cirrhosis (71). Stimulation of hepatic stellate cells (HSCs) is a pivotal event in the fibrosis of hepatocytes and probably contributes to the pathogenesis of cirrhosis (72). Stimulated Kupffer cells can damage hepatocytes and activate HSCs (73). Repeated courses of hepatocyte death and regeneration of these cells contribute to the pathogenesis of liver cirrhosis. In addition, several cytokines play crucial roles in HSC stimulation and activation of fibrogenesis (74, 75). Hepatitis B and C infections and obesity are other risk factors for cirrhosis (76). The majority of hepatocellular carcinoma cases occur after liver cirrhosis; however, cirrhosis itself may happen as a result of an infection, exposure to toxins, immunopathological/autoimmune/allergic reactions, a vascular process, or an inborn metabolism error (77).

**Hepatocellular carcinoma**

Hepatocellular carcinoma (HCC) is the fifth most prevalent cancer in men in the world, and the interaction between the two factors of environment and genetics can probably facilitate HCC development (78). Viruses like HBV and HCV, in addition to NASH, NAFLD, and ingestion of aflatoxin B1 and alcohol, are risk factors for HCC development (Fig. 1) (79, 80). HCC is often associated with liver cirrhosis, which is an outcome of progressive chronic liver diseases by HBV or HCV infection, chronic hepatitis, or autoimmune hepatitis (81). Early diagnosis has led to first-stage treatment and has also significantly increased the survival rate of patients (82-84). Hepatitis B virus infection is the most common cause of HCC in the world and in Iran (85). HBV is more prevalent in some regions of the world such as Asia (86, 87). Patients who suffer chronic HBV infection for a longer duration and/or have less accessibility to diagnostic facilities and prompt anti-viral therapies are at a higher risk for HCC (88). Reports have revealed that infection with HCV is responsible for an increase in the number of cases of HCC among US veterans (89). Additionally, occult HBV or HCV infections, obesity, insulin resistance syndrome, and diabetes should be considered as probable risk factors for HCC in the world today (90). Diabetes and obesity indirectly increase the hazard of HCC by increasing the risk of NAFLD and cirrhosis. The process of inflammatory cascade might be induced because of insulin resistance (91). 

**Diagnosis and preventing of liver diseases**

Liver complications may progress to end-stage liver diseases, such as cirrhosis and/or HCC (92). End-stage complications can be prevented by widespread and precise screening and prompt treatment for chronic liver diseases. Screening for chronic liver disease can be accomplished with the measurement of transaminase concentrations, ultrasonography, and some invasive methods (93). However, using noninvasive methods to screen for liver diseases is more favorable. Liver diseases can be verified by liver biopsy, but this examination is invasive and may not be routinely accomplished (94). Serum liver enzymes such as alkaline phosphatase (ALP), alanine aminotransferase (ALT), Gamma-glutamyl transferase (GGT), and aspartate aminotransferase (AST) can be routinely tested (95). ALP and GGT evaluation might reveal cholestatic complications, while increased levels of ALT and AST might imply the possibility of hepatocellular injuries (96). Albumin and bilirubin are also two important liver function biochemical markers. Bilirubin can specifically increase during liver disease, but the sensitivity of the bilirubin test is not high enough and bilirubin level escalation often occurs in late phases of liver diseases (97). Albumin is used to evaluate the liver’s biosynthetic activity and capability, and the hepatic synthesis of albumin will decrease in end-stage liver diseases (98). Albumin serum levels might drop in cases with disorders like malabsorption, nephrotic syndrome, or protein-losing enteropathy or even malnutrition. Serum ALT activity is the most commonly utilized biochemical marker for liver injury; however, much clinical research has demonstrated that ALT levels do not always correlate well with liver histopathological changes, and ALT activities may be altered in other clinical disorders, such as polymyosistis or skeletal muscle injury. Consequently, the need for more specific and authentic markers of liver injury continues to exist (99).

**MiRNAs in liver diseases**

MicroRNAs (miRNA) are small regulatory and noncoding RNAs that play an essential role in balancing cellular gene expression at post-transcriptional levels (100). MiRNAs can modulate gene expression in a variety of biological process such as proliferation, apoptosis, and differentiation (101). MiRNAs also can regulate viral replication and pathogenesis in a number of different ways which include facilitation, activation of immune responses, epigenetic modulation, and several other effects (102). They bind to the target messenger RNAs (mRNA) and regulate gene expression, so possibly resulting in the inhibition of translation or reduction in the stability of the mRNA, both of which can cause decreased expression of a target protein (103). Each miRNA can control and modulate several genes, and most mRNA targets contain multiple miRNA binding sites (104). Variations in the sequence of a mature miRNA can result in the elimination or changes in binding affinity to target mRNAs. In addition, mutations in target mRNAs are a possible mechanism that may lead to altered expression in some diseases (105). MicroRNAs have been considered a promising candidate for the next generation of noninvasive, diagnostic biomarkers because of the strong correlation between microRNA expression patterns and the progression of several diseases (106). Most miRNA sequences are usually conserved among that species, and some miRNA expression is tissue-specific or highly specific to distinct biological or developmental phases (107). One of the main miRNAs in the liver is miR-122, the expression of which might be dysregulated in HBV and HCV chronic infections. Recent findings have indicated that the miR-122 levels may be linked to the severity of these diseases. The relative quantification of miRNA levels can be easily performed using the real time qPCR technique, which utilizes precise signal amplification (108). Therefore, miRNA quantification can be considered a sensitive, specific, robust, and noninvasive method for assessing disease progression (109). There are several serious challenges facing the effective employment of miRNAs as circulating biomarkers, including inadequate standardized methods which may lead to bias in results interpretation (110). Sample gathering and storage variations, appropriate and effective nucleic acid extraction, miRNA quality and quantity examination, and all other pre-amplification steps are important, technically challenging factors. Variability is another problem which makes difficult the cross comparison of studies published from different laboratories. Three platforms are used for miRNA quantification: microarrays, quantitative real-time PCR, and next-generation sequencing (NGS) (111). Some miRNAs can be deregulated in multiple disorders and in different cancers (112). Some studies have suggested that miRNA can regulate host genes (113). Overall, according to the miRNA potential and based on what has been said, they can be used to determine the susceptibility to developing a disease, measure its progress, or predict prognosis (114). 

Some studies have been done on different miRNAs. Hui Zhang et al. worked on three groups: chronic hepatitis B (CHB) patients, healthy controls, and patients with NASH. They compared the expression levels of 34 miRNAs between these groups. Their results showed that the median levels of miR-122, -572, -575, and -638 were significantly higher while miR-744 was significantly lower in CHB compared with the controls. The levels of miR-122, -572, and -638 were also higher, while the level of miR-744 was lower in CHB than in NASH, although the difference between them was not as significant as that between CHB and controls. Further analysis revealed a probable correlation between these miRNA expression levels and liver function parameters. Serum levels of miR-122, -572, -575, -638, and -744 are deregulated in patients with CHB or NASH, and as a final conclusion, the levels of these miRNAs can serve as potential biomarkers for liver disease caused by NASH and CHB (115). Gui et al. performed miRNAs profiling from HCC/liver cirrhosis patients’ serum pools using qPCR-based TaqMan MicroRNA quantification arrays in comparison with healthy controls. They checked five miRNAs: miR-885-5p, miR-574-3p, miR-224, miR-215, and miR-146a, and found higher levels of miR-885-5p in sera from patients with HCC, liver cirrhosis, and CHB (116). A wide number of studies have shown that HBV infection modulates levels of different host miRNAs, but only a few reports have established the exact role of viral proteins in these modulations. Hepatitis B x protein (HBx) plays a particular role in the regulation of host miRNAs among all viral proteins. Wang et al. found that HBx transcript had significant effects on miR-15a/miR-16-1 suppression, even in the absence of HBx protein. This result confirmed the principal role of the HBx in the modulation of miRNA levels. In addition, the association of HBx protein with miR-29a up-regulation, which in turn enhances cell migration by targeting PTEN in hepatoma cell lines, was determined. DNA methyl transferase3A is also targeted by HBx, ensuring high DNA methylation and managing miR-101 down-regulation. Wang et al. showed that HBx transcript can mediate the repression of miR-15a/miR-16-1 directly, without the essential necessity of HBx protein, thus denoting the vital role of the HBx gene in host miRNA regulation (117). Furthermore, Wang et al. showed that the let-7 family miRNAs (including let-7a, let-7b, and let-7c) which control MYC, Ras, HMGA2, and STAT3 expression, can be targeted by HBx, resulting in the modulation of host oncogenesis and immune response. This study revealed that significant changes in various host miRNAs may happen in HBx expressing cells (118). Another research showed that HBx protein can modulate the expression of tumor suppressor miR-145 and onco-miRs miR-21 and miR-222 in malignant hepatocytes differentially (119). In addition, during chronic hepatitis B progression, miRNA-146a can be up-regulated by HBx protein, and the complement factor H might be down-regulated (120). A profile analysis of miRNAs consisting of miR-122, miR-638, miR-575, and miR-572 was proposed by Zhang et al. with a considerable diagnostic potential for CHB (121). Another study by Zhang et al., conducted in 2015, demonstrated that expression levels of four miRNAs (miR-134, miR-122, miR-629-5p, and miR-424-3p) were up-regulated during HCV infection (122). Another study reported the dysregulation of 106 out of 768 miRNAs in the serum of HCV-infected patients and it further demonstrated that expression levels of four miRNAs (miR-134, miR-134, miR-424-3p, and miR-629-5p) were up-regulated during HCV infection (123). It is assumed the miRNAs that have a role in lipid metabolism and homeostasis possibly participate in the pathogenesis of NAFLD and other metabolic disorders; thus, the evaluation of such role can be helpful for disease diagnosis (124). Moreover, miR-122, miR-34a, and miR-16 serum levels were significantly higher in NAFLD patients than in healthy control groups. The miR-122 and miR-34a levels were also closely linked to disease intensity of NASH (125). The miR-192, miR-21, and miR-122 were found to be significantly overexpressed in NAFLD and NASH patients compared to healthy individuals. In addition, miR-192 and miR-122 were also able to discriminate NAFLD from NASH (126). Interestingly, the down-regulated expression of miR-122 could diagnose NASH from simple steatosis differentially (127). Another study demonstrated that serum levels of miR-122 were positively correlated with the intensity of NASH (128). Previous studies have revealed that miRNAs contribute to the activation of hepatic stellate cells (HSCs) and proliferation, thus regulating the progression of liver fibrosis. Particularly, the miR-29 family has emerged as a key regulator of liver fibrosis (129). Another study showed the potential of miR-200b and miR-122 in human patients for initial diagnosis of liver fibrosis (130). Some studies have indicated that miR-122 can play a principal role in controlling cancer cell proliferation, mutagenesis, and vascular invasion; therefore, it can be regarded as a tumor suppressor (131). Another research showed that the levels of miR-224 and miR-122 in plasma can be helpful in the early diagnosis of hepatocellular carcinoma in humans (132). Some studies have reported miR-221, miR-21, and miR-222 up-regulation and miR-145, miR-122a, miR-199a, and miR-223 down-regulation in HCC in comparison with normal human tissue (133, 134). Some miRNAs have been particularly reported as biomarkers for hepatocellular carcinoma, including miR-214, miR-199a-3p, miR-199a-5p, miR-125a-5p, miR-195, miR-139-5p, miRNA-1269, miR-139-3p, miR-224-3p, miR-497, miR-424-3p, miR-452, and miR-130a. Chen et al. demonstrated the relationship between miR-221 and HCC and its potential as a diagnostic marker for HCC. Furthermore, they suggested that HCC patients with up-regulated miRNA-221 expression had a shorter survival time (135). 

A 2019 study by Zhou et al. demonstrated that circulating miR-122 has an important diagnostic significance for the detection of chronic viral hepatitis (136). Ping Xu et al. compared miR-122, miR-125b, and miR-192 with a conventional marker ALT in 326 serum samples and demonstrated enhanced levels of MiR-125b in HCV patients. In this study, miR-122 and miR-192 were linked to liver injuries by chemicals; however, a major increase was observed in serum miR-122 in patients with active HBV. Interestingly, in alcohol-associated liver injuries, a major increase in these miRNAs could not be observed. Thus, the evaluation of both miR-122 and miR-125b as prognostic biomarkers of liver injury might be helpful (137). In a 2019 survey, Ghaempoor et al. proposed that miR-625 may be considered as a potential noninvasive biomarker for the diagnosis of liver cirrhosis (138). In 2021, Kim et al. examined and evaluated miR-21-5p, miR 192-5p, miR-151a-3p, and miR-4449 as biomarkers for progression to NASH in NAFLD patients (5). Fründt et al. showed in their 2021 study that exosomes secreted into the plasma differentially expressed miRNAs. Moreover, miR-146, miR-192, and miR-16 are prognostic and diagnostic markers for both HCC and liver cirrhosis (139). Ashrafi et al. found that the tissue levels of hsa-miR-3158-5p, -194-5p, -4449, and -548as-3p were correlated to progression to HCC (140). It was also shown that miRs including miR-122-5p, miR-187-3p, miR-193a-5p, and miR-378d were linked to hepatic steatosis in research by Zhang et al. In addition, miR-122-5p, miR-193a-5p, and miR-193b-3p were strongly correlated to liver fibrosis and can be used as biomarkers of fatty liver disease (Table 1) (141).

**MiRNAs in other diseases**

Whether in liver-related diseases or in other complications including cancers, neurological and autoimmune diseases, miRNAs play significant roles through molecular pathways of complex biological processes, such as regulating cell death, proliferation, and differentiation. For example, in cancer, miRNA expression patterns change at different stages of cancer progression. Some cancer-related miRNAs, including miR-16, miR-15, and let-7, were considered as tumor suppressors; however, other miRNAs such as miR-155 and miR-21 may function as oncogenes (142, 143). MiR-16 and MiR-15 cause cell death in cancerous cells by suppressing the expression of anti-apoptotic gene bcl-2 and let-7 family members, demonstrate anti-cancer properties by suppressing oncogene expression, RAS (144, 145). MiR-155 inhibits MSH gene family expressions in CRC and prevents mismatch repair processes. MiR-21 directly serves as an anti-apoptotic determinant in breast cancer and glioblastomas (146). Some studies have elucidated the miRNA functions in neurodegenerative diseases (ND) such as Alzheimer’s disease (AD) and Parkinson’s disease (PD). It seems that because of the very high expression of BACE1 protein, which plays an important role in the pathogenesis of AD, a decrease in the expression levels of miR-9, miR-29a, and miR-29b-1 occurs (147). In Parkinson’s disease, leucine-rich repeat kinase 2 (LRRK2) also promote the expression of transcriptional factors E2F1 by downregulating the expression of miR-184 and let-7 (148). MiRNAs also play critical roles in inflammation by controlling the nuclear factor kappa beta (NF-κB) related pathways, a key factor in inflammatory responses. Several different surveys have implied that miRNAs perform leading roles in various diseases related to the immune system, such as systemic lupus erythematosus (SLE), multiple sclerosis (MS), type I/II diabetes, and also NAFLD. Studies have also shown the up-regulation of miR-155, miR-34a, and miR-326 in MS lesions, decreasing expression of miR-146a in SLA patients, increased expression of miR-200a, miR-200b, and miR-429 and decreased expression of miR-27, miR-122, and miR-451 in diet-induced NAFLD in experimental animal models (149, 150). One study demonstrated that miR-107 and miR-103 negatively regulate insulin sensitivity and glucose homeostasis in type 2 diabetes by targeting caveolin-1, a tight regulator of insulin receptors (151). In viral infections, viruses are capable of regulating the expression of host cellular miRNAs for their own benefit, although the expression of some cellular miRNAs is not directly controlled by viruses. Nonetheless, maintenance of their intracellular levels is pivotal for some viral infections and replication (For example, high levels of liver-specific miR-122 expression is necessary for HCV replication). The increasing levels of miR-155 in HCV patients revealed that up-regulation of miR-155 may activate the Wnt signaling pathway and contribute in part to HCV-induced hepatocarcinogenesis (152-163). To summarize, in addition to host genetic mutations (164, 165) or pathogens’ genetic variations (166-171) that effect some liver diseases progression and clinical status, microRNAs are important molecular players and worth further investigations. 

**Table 1:** A list of the microRNAs and their changes associated with various liver diseases and clinical conditions

Liver disease	Involved miRNAs	Expression	Ref
HCV infection	▪miR-122,miR-134, miR-122, miR-424-3p,miR-629-5p, miR-125b	-Up	(98),(111), (125)
Chronic hepataitis B (CHB)	▪miR-29a, miR-15a/miR-16-1, let-7a, let-7b, let-7c ▪ miR-122, miR-572, miR-575, miR -638, miR-885-5p, miR-145, miR-21, miR-222, miRNA-146a ▪ miR-744, miR-101	-Deregulate -Up -Down	(89),(98), 105),(106), (107), (108),(109),(110),(124) (125)
Nonalcoholic steatohepatitis (NASH)	▪ miR-122, miR-572, miR-575, miR -638, miR-192, miR-21 ▪ miR-744 ▪ miR-21-5p, miR 192-5p, miR-151a-3p, and miR-4449	-Up -Down -Deregulate	(105),(113), (114),(116)
Nonalcoholic fatty liver (NAFLD)	▪miR-122, miR-34a, miR-16, miR-192, miR-21	-Up	(4,(112),(114), (121-123)
Fibrosis	▪miR-29, miR-200b, miR-122, miR-193a-5p, miR-193b-3p	-Deregulate	(117),(118)
Cirrhosis	▪ miR-885-5p ▪ miR-146, miR 192 and miR-16	-Up -Deregulate	(106),(126)
Hepatocellular carcinoma (HCC)	▪ miR-885-5p, miR-221, miR-21, miR-222 miR-224, miR-122 ▪ miR-145 miR-122a, miR-199a,miR-223 ▪ miR-199a-3p, miR-214, miR-195, miR-139-5p, miR-125a-5p, miR-199a-5p, miRNA-1269, miR- 139-3p, miR-224-3p, miR-497, miR-424-3p, miR-452, miR-130a, miR-146, miR-192, miR-16, hsa-miR-3158-5p, miR-194-5p,miR-4449, miR-548as-3p	-Up -Down -Deregulate	(106),(119) (120),(126) (127)
Autoimmune hepatitis (AIH)	▪miR-21, miR-122, miR-10a, miR-133a, miR-210	-Up	(139), (140), (141)
Primary biliary cirrhosis (PBC)	▪ miR-122, miR-378f, miR-328, miR-299-5p, miR-20a-5p, miR106b-5p, miR-93-5p, miR-122-5p, miR-141-3p, miR-451a, miR-642-3p ▪ miR-4311, miR-4714-3p, miR-122a, miR-26a, miR-223-3p, miR-21-5p, miR-26b-5p, miR-505-3p, miR-197-3p	-Up -Down	(142-145) (146-148)
Primary sclerosing cholangitis (PSC)	▪miR-1281, miR-126 miR-200c, miR-122	-Up	(149-151)

## Conclusions

So far, many studies have been performed on the association of miRNAs with various complications, including liver diseases. The stability, sensitivity, and specificity of miRNA expression in tissues and blood circulation make miRNAs potential biomarkers for the diagnosis and prognosis of several diseases, including viral hepatitis and other liver diseases. The relationship between pattern changes in many miRNA levels under different clinical and pathological conditions have been extensively studied. However, there is still a long way to go before miRNAs can reach the clinic as reliable and definitive diagnostic markers. The serum profiling of miRNAs in infectious and non-infectious liver diseases may be introduced as a non-invasive, accurate, and at the same time holistic approach in medical practice in the future.

## Conflict of interests

The authors declare that they have no conflict of interest.
